# Salen-Mn compounds induces cell apoptosis in human prostate cancer cells through promoting AMPK activity and cell autophagy

**DOI:** 10.18632/oncotarget.21138

**Published:** 2017-09-21

**Authors:** Xiaoshuang Tang, Jing Jia, Feng Li, Wei Liu, Chao Yang, Bin Jin, Qi Shi, Xinyang Wang, Dalin He, Peng Guo

**Affiliations:** ^1^ Department of Urology, The First Affiliated Hospital of Xi’an Jiaotong University, Xi’an, Shaanxi, China; ^2^ Department of Urology, The Second Affiliated Hospital of Xi’an Jiaotong University, Xi’an, Shaanxi, China; ^3^ Key Laboratory for Tumor Precision Medicine of Shaanxi Province, Xi’an, Shaanxi, China; ^4^ Oncology Research Lab, Key Laboratory of Environment and Genes Related to Diseases, Ministry of Education, Xi’an, Shaanxi, China

**Keywords:** salen-Mn, prostate cancer, cell apoptosis, AMPK, autophagy

## Abstract

Currently only docetaxel has been approved to be used in the chemotherapy of prostate cancer and new drugs are urgent need. Salen-Mn is a novel type of synthetic reagent bionic and exerts remarkable anticancer activities. However, the effect of Salen-Mn on human prostate cancer has not been elucidated yet. In this study, we found that treatment of PC-3 and DU145 human prostate cancer cells with Salen-Mn inhibited cell growth in dose and time dependent manner. Moreover, Salen-Mn induced cell apoptosis, and increased the expression of apoptotic proteins, such as cleaved caspase-3, cleaved PARP, and Bax, in PC-3 and DU145 prostate cancer cells. Furthermore, we found that Salen-Mn induced expression of LC3-I/II, which is protein marker of cell autophagy, in both dose and time dependent manners; in addition, Salen-Mn increased the phosphorylation of AMPK, suggesting that Salen-Mn increase cell autophagy through activating AMPK pathway. On the other hand, when PC-3 and DU145 cells were treated with Salen-Mn and 3-MA, an inhibitor of cell autophagy, the inhibitory effect of Salen-Mn on cell growth and the induction of apoptotic proteins were decreased. In addition, we found that Salen-Mn inhibited the growth of PC-3 cell xenografts in nude mice. In summary, our results indicate that Salen-Mn suppresses cell growth through inducing AMPK activity and autophagic cell death related cell apoptosis in prostate cancer cells and suggest that Salen-Mn and its derivatives could be new options for the chemical therapeutics in the treatment of prostate cancer.

## INTRODUCTION

Prostate cancer is one of the most common malignancies in male in the USA [1]. According to the epidemiology statistics of global cancer from World Health Organization (GLOBOCAN 2008), the morbidity from prostate cancer in 2008 was ranked second, accounting for 14% of all cancer types in male [2]. Androgen deprivation therapy (ADT) is the first line therapy for most first time diagnostic prostate cancer patients; however, despite initial response rates of 80-90%, patients will progress to castration-resistant prostate cancer (CRPC) [3, 4] , and until now only a few drugs are effective and approved for the treatment of CRPC, such as docetaxel and new generation anti-androgen receptor drugs (abiraterone and enzalutamide). Most patients will be resistant to these drugs soon after the application of them, therefore, it is urgent to develop new treatment regimens for the treatment of prostate cancer.

Cis-diamminedichloro platinum (II) (cisplatin) and cis-diammine (cyclobutane-l, l-dicarboxylato) platinum(II) (carboplatin) are the first transition metal-based anti-tumor drugs in clinical use [5-7]. The platinum based anti-tumor agents have enormous impact on current cancer therapy, however, in order to overcome clinical problems associated with the relatively limited activity of platinum based agents against the broad spectrum of human malignancies, acquired resistance, and side effects, novel non-platinum metal-based anticancer complexes have been and are being developed [5-7]. Salen, N, N’-bis (salicylidene)-ethylene diamine, which is synthetically accessible as it is inexpensive and nontoxic [8], is a promising candidate for this purpose. In this respect, transition Salen-Mn metal complexes are of considerable research interest because of their structural diversity [9]. Fe (III) salen has been reported to induce cell death in breast cancer cell lines *in vitro* [10], suggesting that salen compounds may have anti-tumor properties, although the mechanism by which they induce cell death is unclear. Oxidative stress exerted by redox active metals like Mn may be responsible for DNA/RNA damage *in vitro*, as has been suggested [11, 12]. However, whether Salen-Mn can induces the death of prostate cancer cells and the underlying mechanism are still unknown.

A novel type of Mn (III) salen derivative, which contains amino acid residues and is easier to enter tumor cells, has been synthesized by our collaborators recently [8] and we identified its effect on the proliferation of prostate cancer cells. Moreover, we further detected whether Salen-Mn induced cell apoptosis of prostate cancer cells and whether the cell death is related cell autophagy. Finally, we determined whether Salen-Mn suppresses the growth of prostate cancer cell xenograft in nude mice.

## RESULTS

### Salen-Mn inhibits the growth of PC-3 and DU145 prostate cancer cells

**Figure 1:**
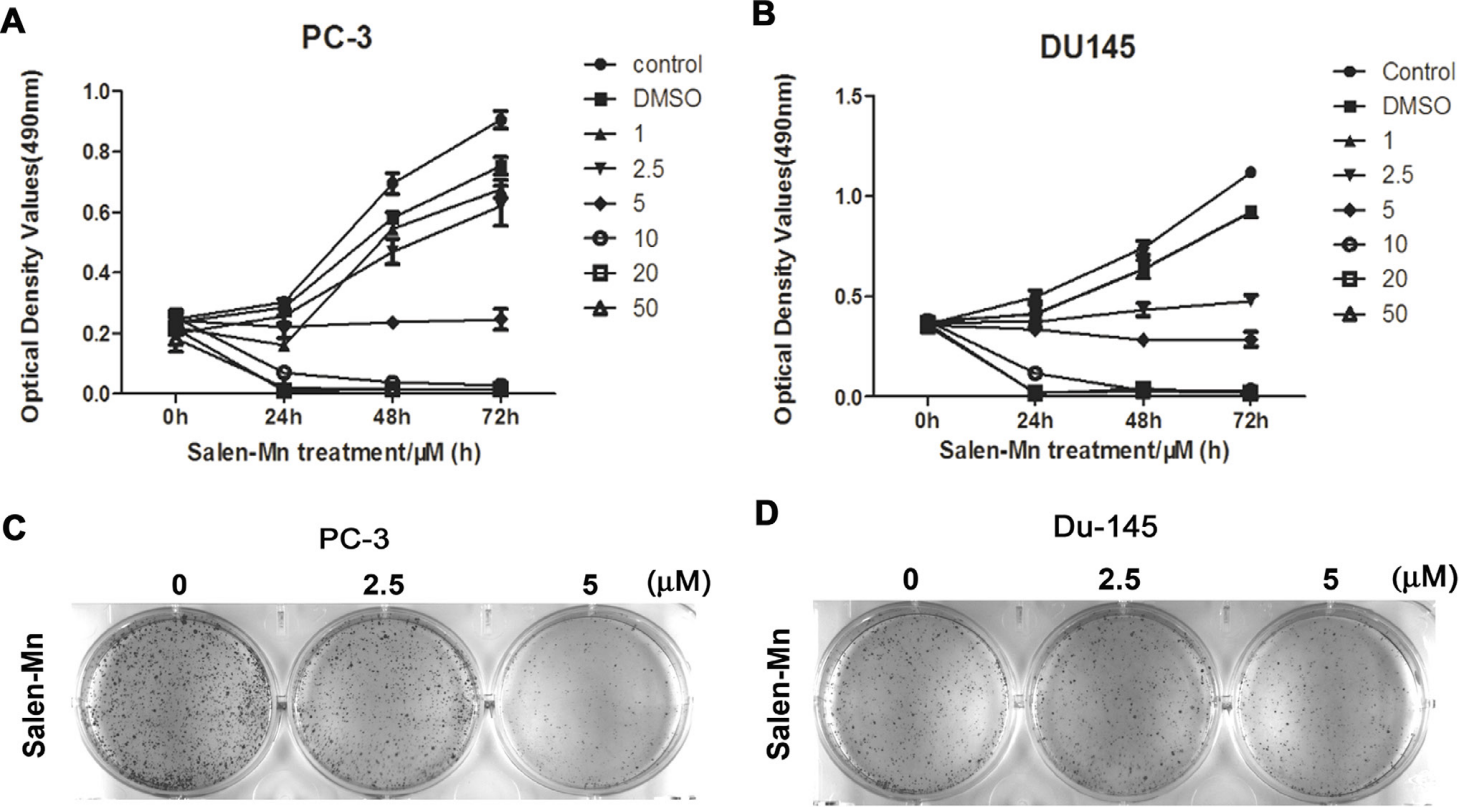
The inhibitory effects of Salen-Mn on proliferation of PC-3 and DU145 prostate cancer cells PC-3 **(A)** and DU145 **(B)** cells were treated with indicated concentrations of Salen-Mn for 24 h, 48 h and 72 h as measured by MTT assay. Each assay was performed in triplicate. The data represents mean ± S.D. C and D, Salen-Mn suppressed the colony formation activity of PC-3 **(C)** and Du145 **(D)** cells. Cells were treated with indicated doses of Salen-Mn for 7 days.

Firstly, we investigated the effect of Salen-Mn on the growth of prostate cancer cells and found that Salen-Mn inhibited the growth of PC-3 and DU145 cells in dose- and time-dependent manners by MTT assay (Figure 1B&1C). Since Salen-Mn treatment at 10, 15, 20, and 30 μM showed very strong inhibitory effect on the proliferation of DU145 and PC-3 cells, 2.5, 5.0, and 10.0 μM were chosen as the representative concentrations for the subsequent *in vitro* treatment of Salen-Mn in prostate cancer cells. Meanwhile, cell colony formation was also obviously inhibited by Salen-Mn treatment in PC-3 and DU145 cells (Figure 1). These results indicate that Salen-Mn can inhibit the growth of prostate cancer cells.

### Salen-Mn induces apoptosis in PC-3 and DU145 prostate cancer cells

**Figure 2:**
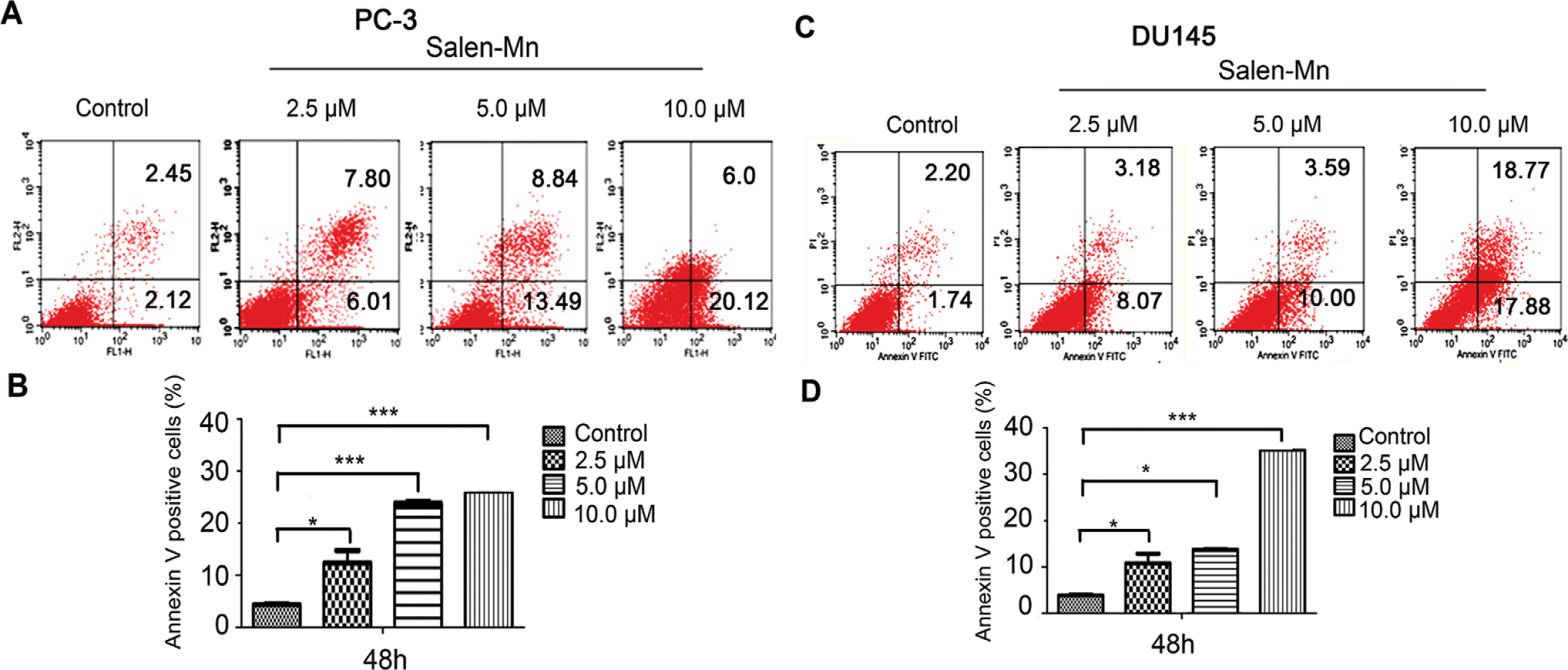
Salen-Mn induced cell apoptosis in prostate cancer cells PC-3 **(A)** and DU145 **(C)** cells were treated with indicated concentrations of Salen-Mn for 48 h and cell apoptosis was detected by cell flow cytometry analysis combining with annexin-V/PI staining. Percentages of annexin-V staining positive cells, which represent apoptosis cells, in PC-3 **(B)** and DU145 **(D)** were qualified. Data represents mean ± S.D.

**Figure 3:**
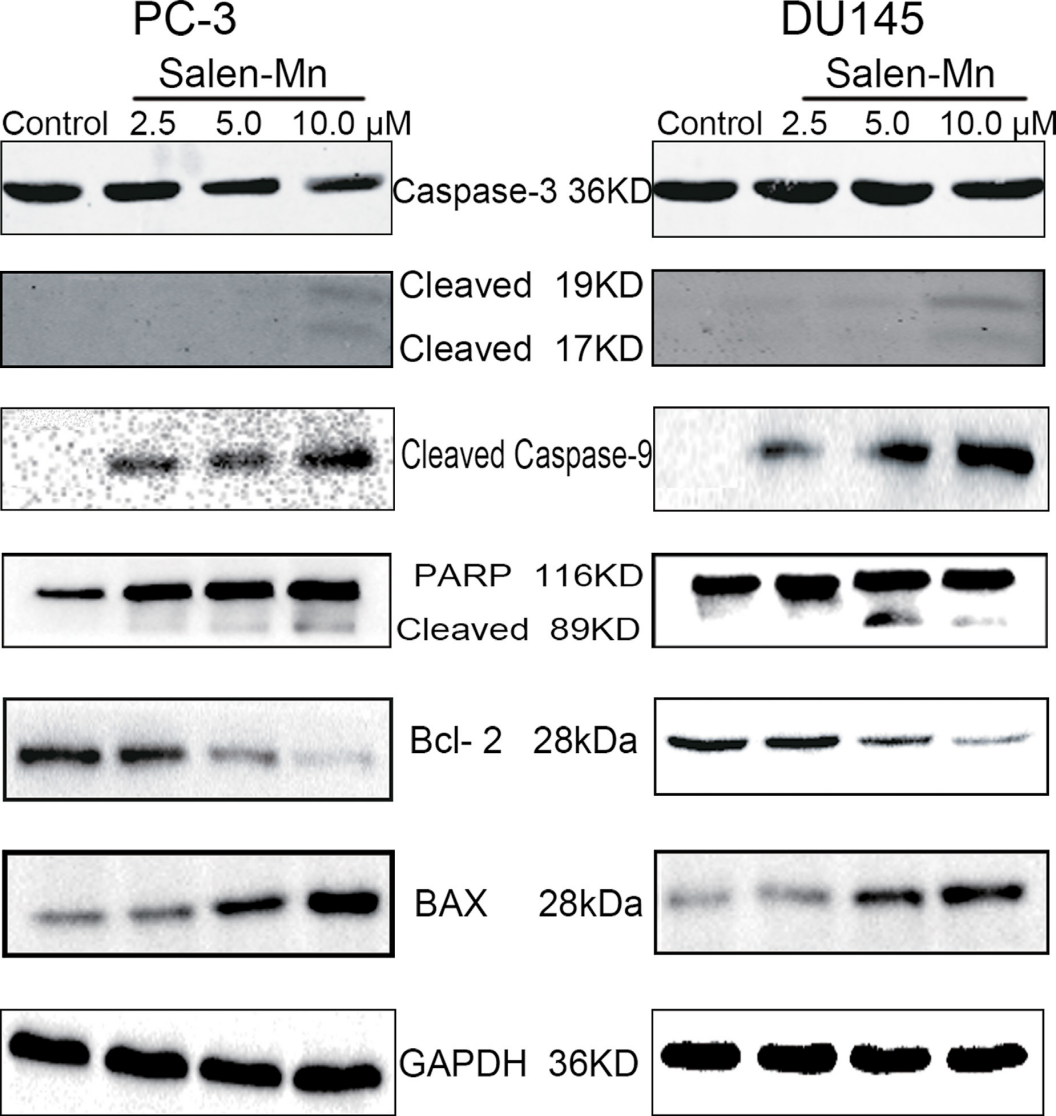
Salen-Mn induced cell apoptosis related protein expression in prostate cancer cells PC-3 and DU145 cells were treated with indicated concentrations of Salen-Mn for 48 h and protein expression was detected by western blotting analysis. GAPDH was used as a loading control.

Since a significant inhibitory effect of Salen-Mn on PC-3 and DU145 cells was observed, we further detected whether Salen-Mn could induce apoptosis in prostate cancer cells by annexin V and PI double staining. As shown in Figure 2A and 2B, Salen-Mn treatments at 2.5, 5, and 10 μM for 48 h resulted in 13.81%, 22.33% and 26.12% of apoptotic cells in PC-3 cells, respectively, and the baseline apoptosis of the vehicle control cells was 5.08% (*P<*0.05), as detected by flow cytometry. Similar effects were observed in DU145 cells (Figure 2C&2D). These results indicated that Salen-Mn could induce apoptosis in human prostate cancer DU145 and PC-3 cells in a dose-dependent manner. Moreover, as shown in Figure 3, Salen-Mn could activate the cleavage of caspase-3 (17 kDa and 19 kDa) and PARP (89 kDa) in PC-3 and DU145 cells in a dose-dependent manner as detected by western blot analysis, indicating that Salen-Mn triggered caspase cascade in the induction of cell apoptosis. In addition, Salen-Mn decreased the protein level of Bcl-2 and increase the expression of Bax. Taken together, Salen-Mn modulates the expression of apoptosis related molecules and induces the apoptosis of prostate cancer cells.

### Salen-Mn induces autophagy in prostate cancer cells

**Figure 4:**
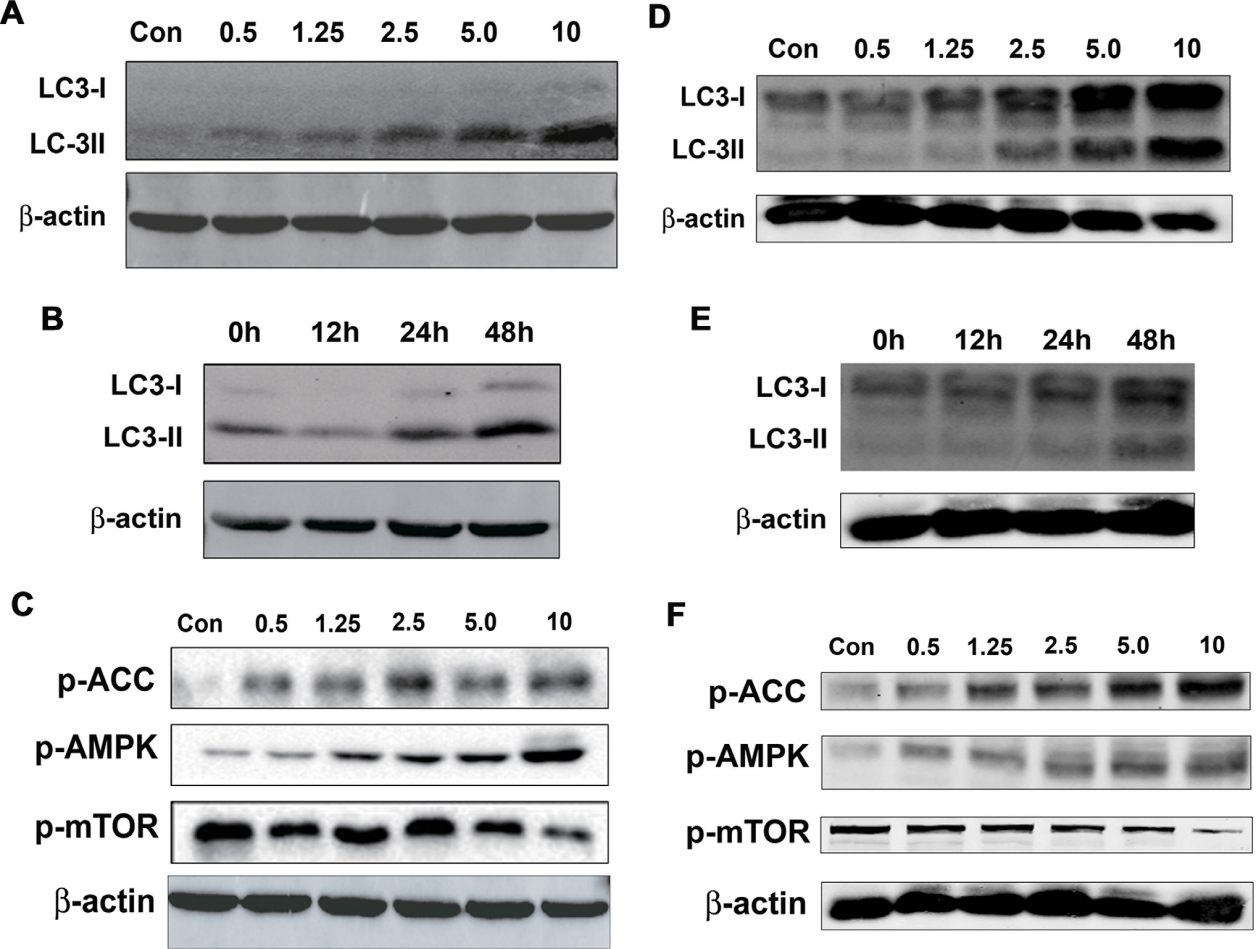
Salen-Mn promoted activity of AMPK pathway and increased cell autophagy in prostate cancer cells **(A**&**D)** Salen-Mn increased expression of LC3-I/II in a dose-dependent manner in PC-3 (A) and DU145 (D) cells, as detected by western blotting analysis. Cells were treated with indicated concentrations of Salen-Mn for 48 h. B&E, Salen-Mn increased expression of LC3-I/II in a time-dependent manner in PC-3 **(B)** and DU145 **(E)** cells, as detected by western blotting analysis. Cells were treated with 5 μM of Salen-Mn for indicated times. C&F, Saleen-Mn induced the phosphorylation of AMPK and ACC (a downstream molecule of AMPK) and inhibited phosphorylation of mTOR in a dose-dependent manner in PC-3 **(C)** and DU145 **(F)** cells.

Autophagy is an evolutionarily conserved self-eating process, which degrades cytoplasmic proteins and organelles and it is essential for cellular homeostasis [15, 16]. Although autophagy can promote cell survive via degrading unnecessary molecules or organelles to supply materials that is needed for cell metabolism; on the other hand, autophagy may interplay with apoptosis or cell cycle arrest or directly trigger autophagic cell death, which subsequently leads to inhibition of cancers [17-19]. To detect whether Salen-Mn increased cell autophagy, we determined the effect of Salen-Mn on the expression of LC3-I/II, which are molecular markers of autophagy, by western blotting analysis. We found that Salen-Mn induced expression of Lc3-I/II in both dose-dependent and time-dependent manners in PC-3 (Figure 4A&4B) and DU145 (Figure 4D&4E) prostate cancer cells.

AMP activated protein kinase (AMPK), a key energy sensor, which has been implied to affect mTORC1 to regulate autophagy, was then evaluated in PC-3 and DU145 cells treated with different concentrations of Salen-Mn. We found that Salen-Mn significantly increased the level of phospho-AMPK (Thr172) and its target phospho-ACC (Ser79) in a dose-dependent manner in both PC-3 (Figure 4C) and DU145 (Figure 4F) cells, indicating the activation of AMPK signaling by Salen-Mn. In addition, mTOR complex 1 (mTORC1) signaling is critical for autophagy induction. Activated mTORC1 suppressing autophagy, while repression of mTORC1 inducing autophagy [16]. As shown in Figure 4C and 4F, Salen-Mn inhibited the phosphorylation of mTOR in a dose-dependent manner in both PC-3 and DU145 cells. In summary, our results indicate that Salen-Mn could increase cell autophagy in prostate cancer cells, which may be due to the activation of AMPK and the subsequent inhibition of mTOR activity.

### Cell growth inhibition and cell apoptosis induction by Salen-Mn treatment were dependent on cell autophagy

**Figure 5:**
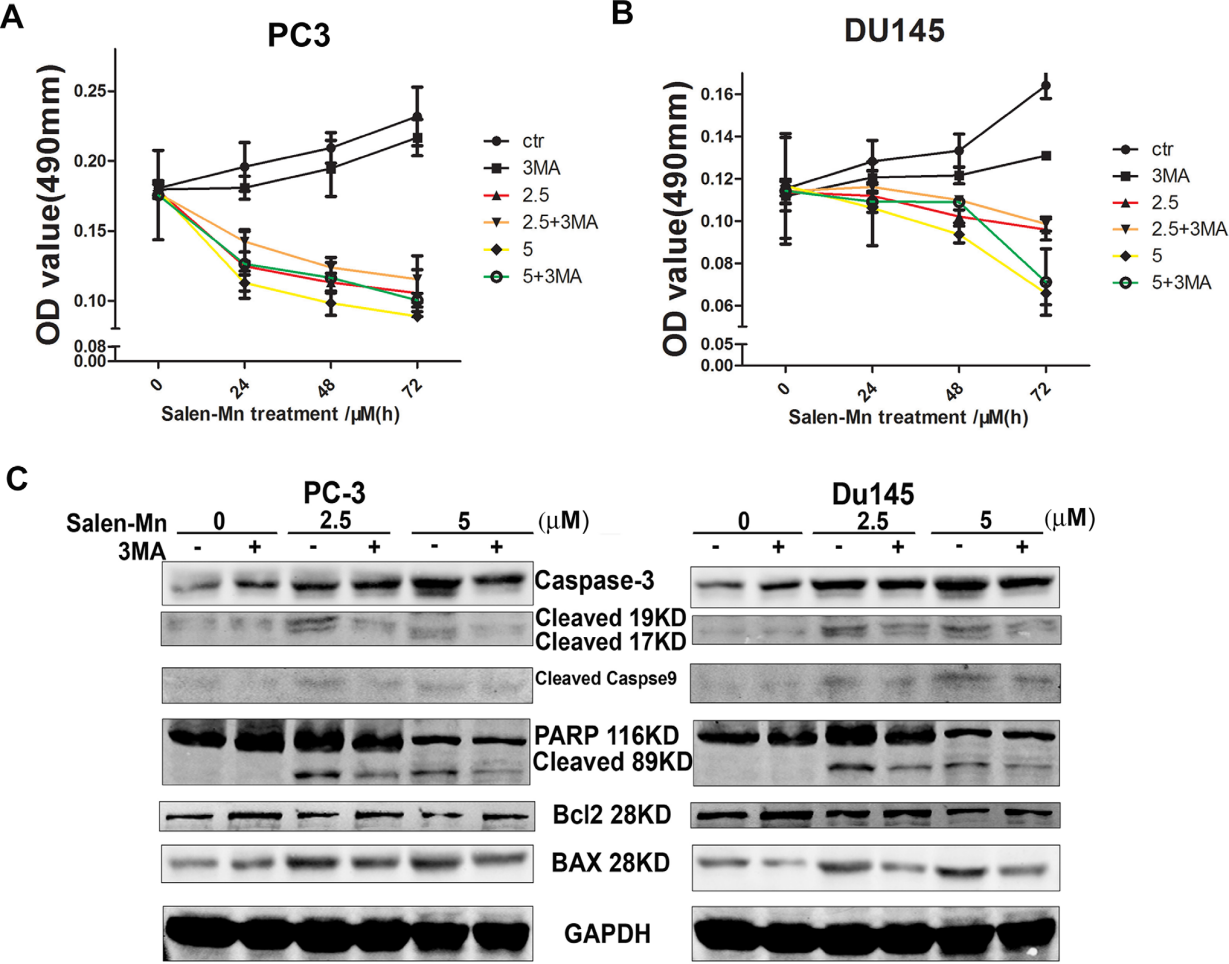
Cell growth inhibition and cell apoptosis induction by Salen-Mn treatment were dependent on cell autophagy **(A**&**B)** Cell autophagy suppression decreased the inhibitory effect of Salen-Mn on PC-3 (A) and DU145 (B) cells, as detected by MTT assay. **(C**&**D)** Cell autophagy suppression decreased the apoptotic protein induced by Salen-Mn in PC-3 (C) and DU145 (D) cells, as detected by detected by western blotting analysis.

To determine whether cell autophagy plays an important role in the cell growth inhibition by Salen-Mn, we treated prostate cancer cells with Salen-Mn and 3-MA, which is as inhibitor of cell autophagy. As shown in Figure 5A and 5B, we found that 3-MA treatment decrease the inhibition of cell growth by Salen-Mn in PC-3 and DU145 cells, though 3-MA itself inhibit cell growth slightly, as detected by MTT assay. On the other hand, 3-MA treatment decreased the induction of apoptotic proteins, such as cleaved caspase-3, cleaved caspase-9, cleaved PARP and Bax by Salen-Mn in both Pc-3 and DU145 cells (Figure 5C&5D). Taken together, our results indicate that autophagic cell death is important in the cell apoptosis induced by Salen-Mn, and autophagic cell death plays an important role in the cell growth inhibition by Salen-Mn as well.

### Salen-Mn inhibits the growth of PC-3 prostate cancer cell xenograft *in vivo*

**Figure 6:**
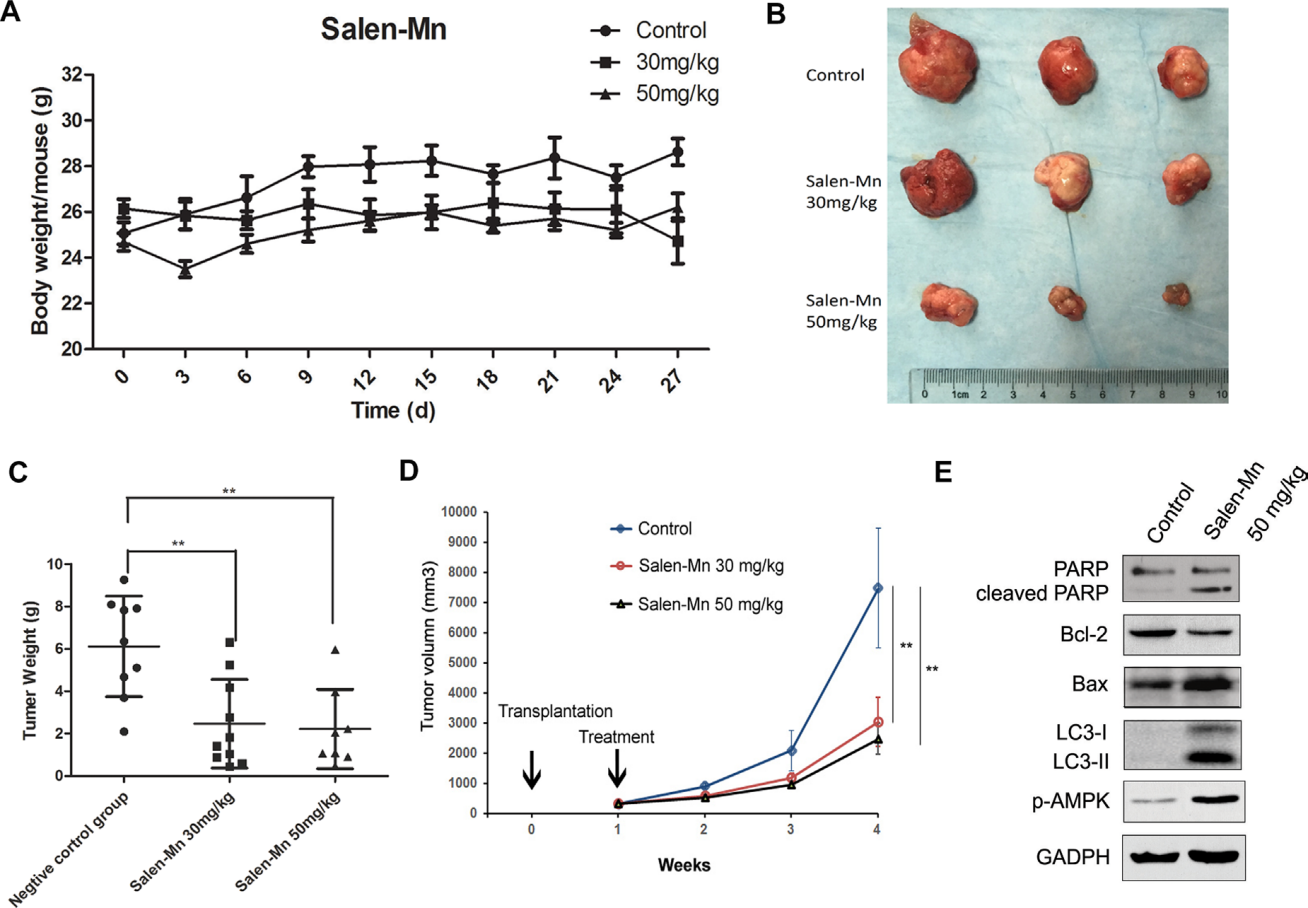
Salen-Mn inhibits the growth of prostate cancer cell xenograft *in vivo* **(A)** The body weight of mice treated with 30 mg/kg and 50 mg/kg had no significant difference with that of control mice treated with vehicle. **(B)** The PC-3 cell xenografts treated with Salen-Mn were taken from nude mice. **(C)** The tumor weight of the xenografts in mice were significantly decreased by Salen-Mn treatment at both 30 mg/kg and 50 mg.kg when comparing with control group. **(D)** The tumor growth curves of PC-3 cell xenografts in mice treated with vehicle (control) or Salen-Mn. **(E)** Protein expression of apoptosis and autophagy related markers in tumor xenografts were detected by western blot analysis. **:p<0.01.

To confirm the effect of Salen-Mn on the growth of prostate cancer cells, we injected PC-3 cells into nude mice and applied Salen-Mn on the mice. The body weight of mice treated with 30 mg/kg and 50 mg/kg of Salen-Mn had no significant difference with that of control mice treated with vehicle (Figure 6A). On the other hand, the tumor size, tumor weight and tumor growth curve of the PC-3 cell xenografts in nude mice were significantly decreased by Salen-Mn treatment at both 30 mg/kg and 50 mg/kg when comparing with control group treated with vehicle (Figure 6B, 6C&6D). Moreover, Salen-Mn treatment (50 mg/kg) obviously increased expression of cleaved PARP, Bax and decreased expression of Bcl-2 in the xenograft tumor tissues comparing with control group treated with vehicle, suggesting that Salen-Mn also elevated cell apoptosis *in vivo* (Figure 6E). Consistently, Salen-Mn increased expression of p-AMPK and LC3-I/II, suggesting that Salen-Mn activated AMPK pathway and induced cell autophagy in the xenograft tumors (Figure 6E). These results indicate that Salen-Mn suppresses the growth of prostate cancer xenografts and increased cell autophagy and cell apoptosis *in vivo*.

## DISCUSSION

In the present study, we found that Salen-Mn inhibited cell growth, induced cell apoptosis, and increased the expression of apoptotic proteins, in PC-3 and DU145 prostate cancer cells. Furthermore, Salen-Mn increased cell autophagy and phosphorylation of AMPK, and decreased the phosphorylation of mTOR, suggesting that Salen-Mn increase cell autophagy through activating AMPK pathway. On the other hand, inhibitor of cell autophagy decreased the inhibitory effect of Salen-Mn on cell growth and the induction of apoptotic proteins, indicating that the autophagic cell death plays an essential role in the Salen-Mn-inducing cell apoptosis. In addition, we confirmed that Salen-Mn inhibited the growth of PC-3 cell xenografts in nude mice, which is the first report on the anticancer function of Salen-Mn *in vivo*. In summary, our results indicate that Salen-Mn suppresses cell growth through inducing activity of AMPK pathway and autophagic cell death related cell apoptosis in prostate cancer cells.

Cell autophagy induced by anticancer reagents has been widely studied in various cancer cell models [13] and abundant evidence shows that autophagy plays dual roles in carcinogenesis and anticancer therapies which depends on the contents degraded by it. Autophagy degrades unfolded or aggregated proteins and organelles to maintain intracellular metabolic homeostasis and thus facilitates the cancer cells to resist to chemotherapy and radiotherapy [14]. On the other hand, it may degrade the anti-apoptotic protein Bcl-2 to trigger cell apoptosis [15]. Thus, the effects of drug-inducing autophagy are complexed. If the autophagy does not lead to cell death, such as autophagic apoptosis, it will help cells to survive the stress caused by drugs and protect cells from death and result in cell resistance to the drug. In our study, we first document that Salen-Mn induces autophagy in PC-3 and DU145 prostate cancer cells. Inhibition of autophagy by 3-MA significantly attenuated or enhanced the anti-proliferation effects of Salen-Mn, suggesting the anti-proliferation functions of autophagy induced by Salen-Mn. Considering that Salen-Mn promotes cell apoptosis in prostate cancer cells, our results indicate that Salen-Mn increased autophagic death, which leads to cell apoptosis and cell growth inhibition.

Several critical molecules and pathways have been demonstrated to regulate autophagy progress [16-18] and among which the best characterized is AMPK/mTOR pathway [19, 20]. Ulk1/2-Atg13-FIP200 complex is considered to be the initial step of autophagosome biogenesis and mTORC1 crosstalk with it via direct interaction between raptor and Ulk1/2. Active mTOR1 phosphorylates Atg13 and Ulk1/2, thereby suppressing Ulk1/2 kinase activity [21]. The network of AMPK inducing autophagy is more complicated. AMPK mediates the activity of mTORC1 *via* phosphorylating Raptor and TSC2, two negative regulator of mTORC1, to induce autophagy [22, 23]. Meanwhile, AMPK could directly interact with Ulk1 and positively regulate its activity through AMPK-dependent phosphorylation, further enlarges the range of possibilities for AMPK to induce autophagy [24]. Our further mechanistic studies revealed that the autophagy induction by Salen-Mn was mTOR-dependent and regulated by AMPK. Salen-Mn strongly inhibited the activation of mTOR pathway but activated the AMPK pathway. This is the first report that Salen-Mn can activate AMPK, suggesting that Salen-Mn could be used not only in the treatment of cancer but also other diseases such as diabetes.

Salen-Mn compounds, which are a kind of metallo-drugs, have recently been explored for their anticancer properties [12] [11]. Salen-Mn complexes possess ability to bind with free-radicals like hydrogen peroxide decomposition, superoxide anion (O2^-^) dismutase, catalase, water oxidation and ribonuclease reduction, and DNA and proteins. It has been reported that Salen-Mn (III) has strong antioxidant activity [25], moreover, it has the DNA binding and cleavage activity [26, 27]. Mn(III)-salen complexes are shown to possess superoxide dismutase (SOD) and catalase activities and are considered as synthetic SOD mimics [28]. Like most of the anticancer agents, Salen-Mn can induce apoptosis in cancer cells, which could be due to DNA damage or antioxidant activity, but the underlying mechanism is not clear. In the present study, we found that Salen-Mn can trigger the activity of AMPK, which leads to cell autophagic death and cell apoptosis. The activation of AMPK may be caused by the interaction between AMPK and Salen-Mn or the SOD like function of Salen-Mn, and we will identify the mechanism in the subsequent study.

In conclusion, we found that Salen-Mn inhibited cell growth of prostate cancer cells *in vitro* and *in vivo*, moreover, Salen-Mn suppresses cell growth through inducing activity of AMPK pathway and autophagic cell death related cell apoptosis. Our results suggest that Salen-Mn and its derivatives could be new options for the chemical therapeutics in the treatment of prostate cancer.

## MATERIALS AND METHODS

### Cell culture and regents

Human prostate cancer DU145 and PC-3 cell lines were from the American Type Culture Collection (Manassas, VA, USA). Cells were cultured in Dulbecco’s Modified Eagle’s medium/1640 supplemented with 10% fetal bovine serum (Gibco, Grand Island, NY, USA) and 1% penicillin-streptomycin, at 37°C, in humidified air containing 5% CO_2_.

Salen-Mn has been synthesized from Xi’an Jiaotong University [8]. Salen-Mn was made into a fine suspension by dissolving the compound in DMSO, which was diluted to desired concentrations in the medium immediately before each experiment. 3-(4,5-dimethylthiazol-2-yl)-2, 5-diphenylSalen-Mnrazolium bromide (MTT) were purchased from Sigma Chemical Co. (St. Louis, MO, USA). 3-methyadenine (3-MA, 189490) was purchased from Merck Millipore (Darmstadt, Germany). Antibodies against cleaved caspase-3, poly (ADP-ribose) polymerase (PARP), Bcl-2, BAX, LC3-I/II, p-AMPK, p-ACC, p-mTOR and peroxidase-conjugated secondary antibodies were from Cell Signaling Technology (Beverly, MA, USA). Antibody against glyceraldehyde-3-phosphate dehydrogenase was from Santa Cruz Biotechnology, Inc. (Santa Cruz, CA, USA). The enhanced chemiluminescence (ECL) detection system was obtained from Amersham Life Science, Inc. (Arlington Heights, IL, USA).

### 3-(4,5-Dimethylthiazol-2-yl)-2,5-diphenyltetrazolium bromide (MTT) assay

Cell viability and growth rate were measured by the3-(4,5-dimethylthiazol-2-yl)-2,5-diphenyltetrazolium bromide (MTT) assay, as described previously [29]. Briefly, 5 × 10^3^ normal cultured or transfected cells were seeded in 96-well culture plates, followed by various treatments for the indicated time, and then, cells were washed once and incubated with 0.5 mg/mL of MTT at 37 °C for 4 h. The medium was discarded carefully and 150 μL DMSO were added to solubilize the formazan crystals. Finally, the absorbance was measured for each well at a wavelength of 490 nm using the Microplate Autoreader (Bio-Tek Instruments Inc., Winooski, VT, USA). Independent experiments were repeated in triplicate.

### Colony formation assay

Cells were seeded in 6-well plate (1,000 cells/well) in 2 ml culture medium overnight. In drug treatment group, the medium was changed with fresh medium containing Salen-Mn (2.5 and 5 μM) or vehicle (DMSO) every other 2 days and cells continued to grow for two weeks. Colonies were fixed with 4% paraformaldehyde and stained by crystal violet for 10 min respectively at room temperature. Colonies consisted of more than 50 cells were counted [20].

### Apoptosis assays

Cells were trypsinized and harvested, then stained by Annexin-V following the protocol of apoptosis assay kit (Annexin-V-FLUOS staining Kit, Roche, Mannheim, Germany). Apoptosis was characterized as the percentage of Annexin-V positive cells when examined by flow cytometry.

### Western blot analysis

The procedure was described previously [30]. Briefly, at the end of treatment, cultured cells were washed once with cold PBS and lysed in RIPA buffer (50 mM Tris pH 8.0, 150 mM NaCl, 0.1% SDS, 1% NP-40 and 0.5% sodium deoxycholate) containing protease inhibitors. Approximately 30 μg of protein were separated with 10%-12% SDS-PAGE gel and blotted onto nitrocellulose membranes. The membranes were blocked with 5% skim milk at room temperature for 1 h and then incubated with primary antibodies against GAPDH (1:10,000, Shanghai Kangchen, Shanghai, China), or other antibodies at 4 °C overnight, followed by a TBST wash and 1 h incubation with horseradish peroxidase-conjugated secondary antibodies at room temperature. Protein bands were visualized by a Molecular Imager ChemiDoc XRS System (Bio-Rad Laboratories, Hercules, CA, USA).

### Tumor xenograft model

Briefly, 2 × 10^6^ PC-3 cells in 200 μL of serum-free RPMI-1640 medium mixed with matrigel (1:1 *v*/*v*) were injected subcutaneously into two flanks of 5-week-old male BALB/c nude mice. After 7 days, tumor-bearing mice were randomly divided into vehicle- , low dosage Salen-Mn (30mg/kg) - and high dosage Salen-Mn (50mg/kg) -treated groups (6 mice per group). For Salen-Mn-treated groups, Salen-Mn in phosphate buffered saline (PBS) was injected intraperitoneally every two days for 30 days. The vehicle group received PBS only. At the end of the experiment, mice was sacrificed, and tumors were weighed and then preserved in liquid nitrogen. Tumor volume was calculated according to the formula 0.5 × length × width× thickness. Animal care and protocols were approved by the Institutional Animal Care and Use Committee of Xi’an Jiaotong University on March 19, 2015, and the permit number was SCXK2015-0061.

### Statistical analysis

All statistical analyses were performed using SPSS 17.0 (SPSS Inc, Chicago, IL, USA). Statistical differences among the control and various treatment groups was compared using one-way analysis of variance, followed by Dunnett’s *t*-test for multiple comparisons. Student’s *t*-test (two-sided) was used for comparisons involving only two groups. *P<*0.05 was considered statistically significant.
